# Therapeutic targeting of Aurora A kinase in Philadelphia chromosome-positive ABL tyrosine kinase inhibitor-resistant cells

**DOI:** 10.18632/oncotarget.25985

**Published:** 2018-08-21

**Authors:** Seiichi Okabe, Tetsuzo Tauchi, Yuko Tanaka, Kazuma Ohyashiki

**Affiliations:** ^1^ Department of Hematology, Tokyo Medical University, Tokyo, Japan

**Keywords:** chronic myeloid leukemia, Aurora kinase, ABL tyrosine kinase inhibitor, cell cycle, T315I mutation

## Abstract

Abelson murine leukemia viral oncogene homolog (ABL) tyrosine kinase inhibitors (TKIs) have been shown to be effective for treatment of chronic myeloid leukemia (CML) and Philadelphia chromosome-positive (Ph+) acute lymphoblastic leukemia patients. However, resistance to ABL TKIs can develop as a result of breakpoint cluster region-ABL point mutations. Aurora kinases regulate many processes associated with mitosis. In this study, we investigated whether inhibiting Aurora kinase can reduce the viability of Ph+ leukemia cells. Treatment with the Aurora kinase A inhibitor alisertib blocked Ph+ leukemia cell proliferation and Aurora kinase A phosphorylation; it also induced G2/M-phase arrest and increased the intracellular levels of reactive oxygen species. Combined treatment of Ph+ cells with ABL TKIs and alisertib was cytotoxic, with the fraction of senescent cells increasing in a time- and dose-dependent manner. Aurora A gene silencing suppressed cell proliferation and enhanced ABL TKI efficacy. In a mouse xenograft model, co-administration of ponatinib and alisertib enhanced survival and reduced tumor size; moreover, the treatments were well tolerated by the animals. These results indicate that inhibiting Aurora kinase can enhance the cytotoxic effects of ABL TKIs and is, therefore, an effective therapeutic strategy against ABL TKI-resistant cells, including those with the T315I mutation.

## INTRODUCTION

Chronic myeloid leukemia (CML) is a myeloproliferative neoplasm characterized by cytogenetic aberration, namely, the translocation of the Philadelphia chromosome (Ph) [1], which generates a breakpoint cluster region-Abelson murine leukemia viral oncogene homolog 1 (BCR-ABL1) fusion oncogene that encodes the BCR-ABL oncoprotein [2]. Treatment options for CML include ABL tyrosine kinase inhibitors (TKIs) such as imatinib, which has been shown to be effective in patients [3]. However, resistance to imatinib can develop as a result of BCR-ABL point mutations [4, 5], such as the T315I gatekeeper substitution mutation in the ABL kinase domain [6]. CML patients with T315I mutation exhibit a reduced response to ABL TKIs, including the second-generation drugs nilotinib, dasatinib, and bosutinib [6].

Ponatinib (also known as AP24534) is an oral multi-target third-generation TKI [7] that can overcome imatinib resistance due to T315I mutation. Ponatinib is also effective against heavily pretreated resistant CML and in one-third of patients in the accelerated or blastic phases of the disease [8]. However, in a clinical trial, only 62% of refractory CML patients exhibited a cytogenetic response to ponatinib treatment^8^. Therefore, new therapeutic approaches are needed to overcome ABL TKI resistance and improve the outcome of Ph-positive (Ph+) leukemia patients.

Mitosis is a normal part of the cell cycle [9] that is dysregulated in cancer. Aurora kinases are a family of serine/threonine kinases that are required for mitotic progression [10]. The mammalian genome contains three Aurora kinases (Aurora A, B, and C); Aurora A is essential for mitotic spindle assembly [11] and is overexpressed in cancer cells. Thus, Aurora A is an attractive target for Ph+ leukemia treatment, including cases that are resistant to ABL TKIs [12, 13].

In this study, we investigated whether inhibiting Aurora kinase A can reduce the viability of Ph+ leukemia cells that are resistant to ABL TKIs.

## RESULTS

### Aurora A expression in Ph+ leukemia cells

**Figure 1:**
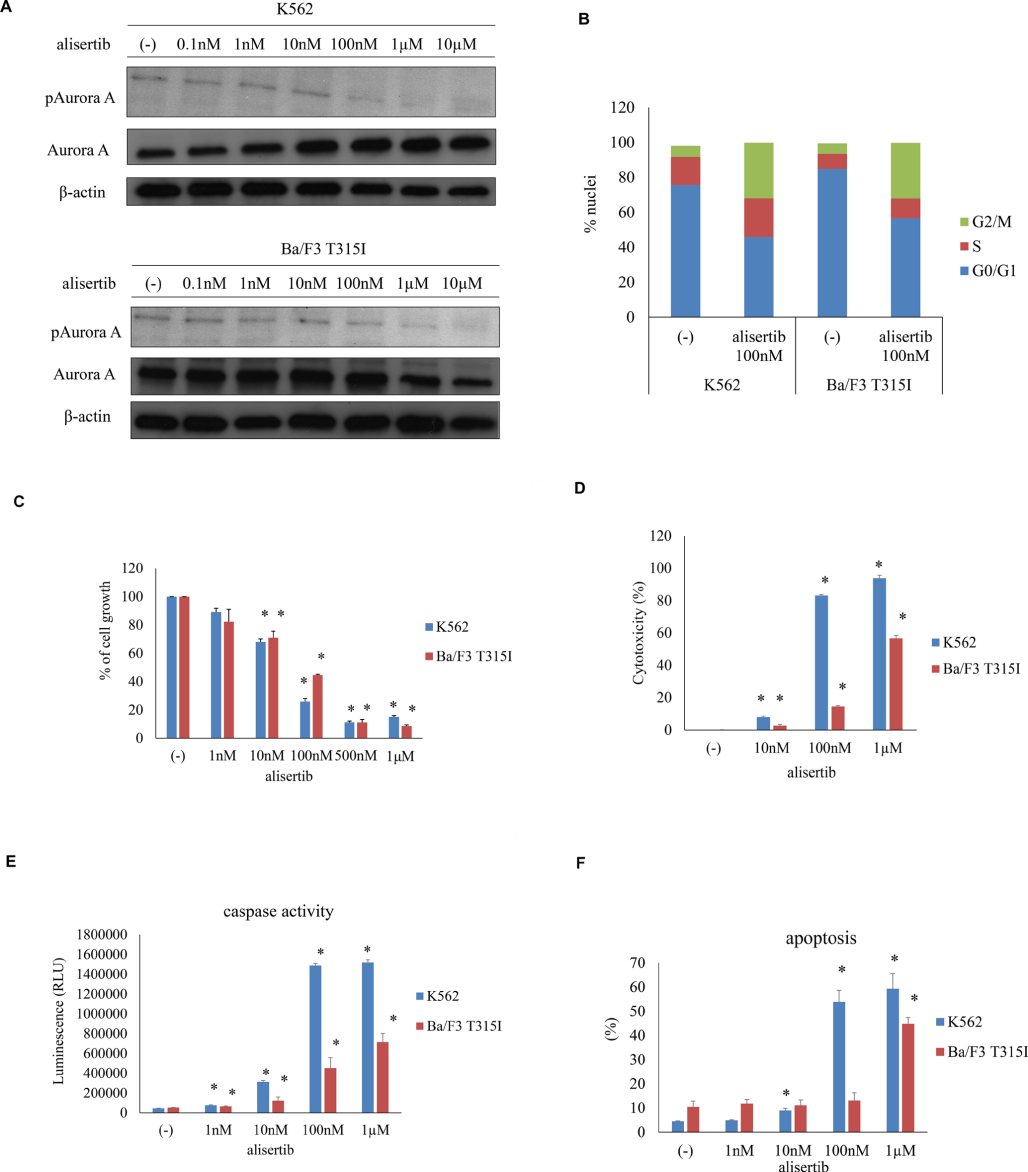
Effect of alisertib on Aurora A activity in Ph+ cells (**A**) Aurora A phosphorylation was examined by immunoblotting, with β-actin serving as a loading control. (**B**) Cell cycle status in alisertib-treated Ph+ cells. (**C**) K562 or Ba/F3 T315I cells were treated with indicated concentrations of alisertib for 72 h, and cell growth was evaluated. (**D**–**F**) effect of alisertib on cell viability and apoptosis, as determined by LDH release (D), caspase activity (E), and flow cytometric detection of apoptotic cells (F). ^*^P < 0.05 vs. control. Results represent the mean of three independent experiments.

Aurora A is essential for normal mitotic spindle formation and centrosome maturation. Aurora A phosphorylation at Thr288 in the catalytic domain increases its kinase activity [14]. We, therefore, examined the phosphorylation level of Aurora A by immunoblotting to determine whether targeting Aurora A can affect mitosis in K562 and Ba/F3 T315I Ph+ leukemia cells. We found that Aurora A was phosphorylated in K562 and Ba/F3 T315I cells and that this was reduced in a dose-dependent manner by treatment with alisertib for 48 h (Figure 1A). It was previously reported that inhibiting Aurora kinase A leads to mitotic arrest [10]. We, therefore, examined cell cycle status by flow cytometry in alisertib-treated Ph+ leukemia cells. After treatment with 100 nM alisertib for 48 h, the G2/M fraction of K562 and Ba/F3 T315I cells was increased (Figure 1B). To determine the effect of alisertib on cell growth, K562, Ba/F3 T315I, ABL TKI resistant K562 cells (K562 imatinib resistant: IR, nirotinib resistant: NR) were treated with various concentrations of alisertib for 72 h. We found that proliferation was decreased in a dose-dependent manner (Figure 1C, Supplementary Figure 1A) and also that the other Aurora A inhibitor, Aurora Inhibitor I, was inhibited in K562 cells (Supplementary Figure 1B). Moreover, cytotoxicity and caspase 3/7 activity were dose-dependently enhanced in the presence of alisertib (Figure 1D, 1E). Consistent with these observations, we observed that alisertib increased apoptosis in K562 and Ba/F3 T315I cells (Figure 1F).

### Efficacy of ABL TKIs and alisertib against Ph+ cells

**Figure 2:**
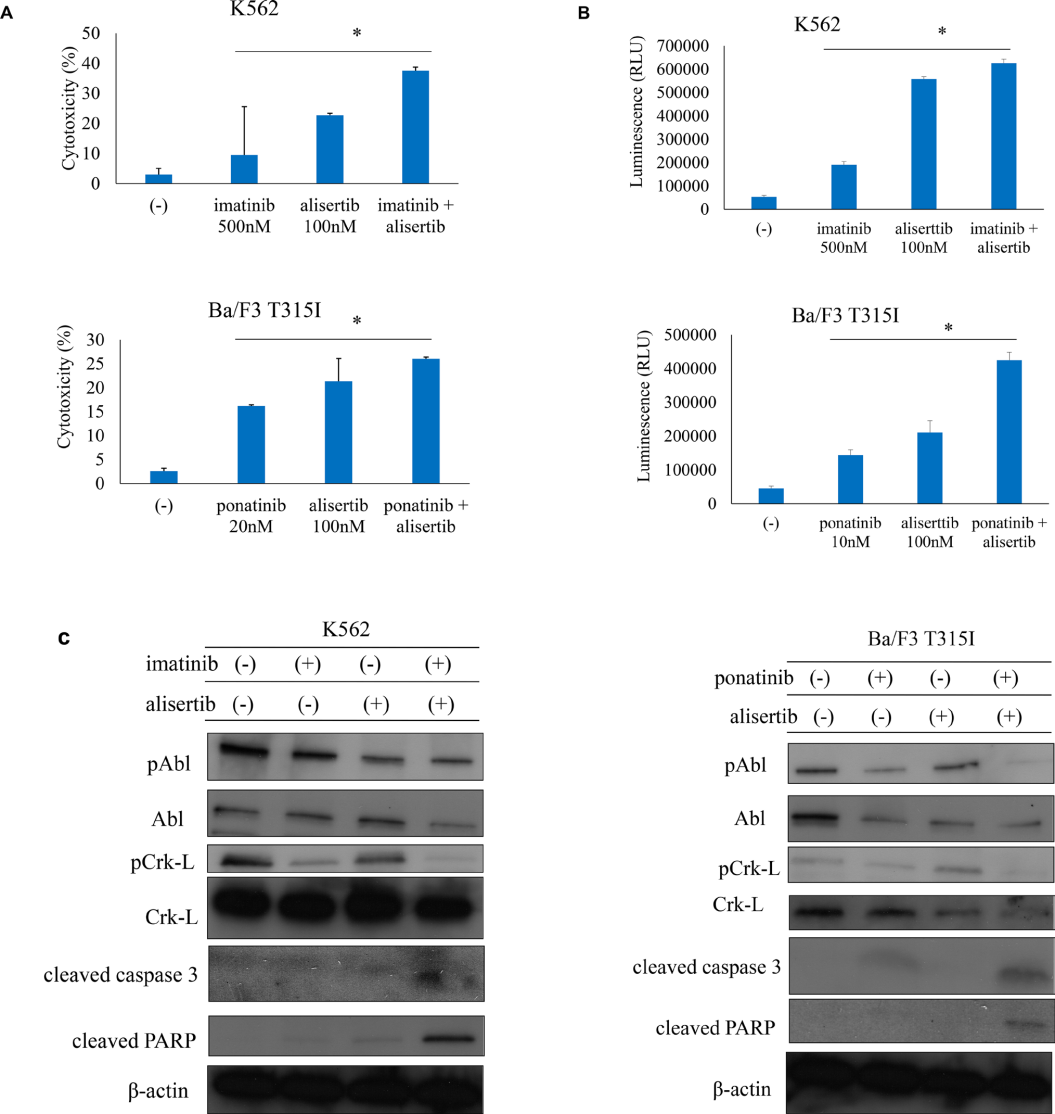
ABL TKIs combined with alisertib induces cytotoxicity in Ph+ cells (**A**, **B**) K562 or Ba/F3 T315I cells were treated with ABL TKIs and/or alisertib for 48 h or 72 h. Cytotoxicity (A) and caspase activity (B) were evaluated. (**C**) K562 or Ba/F3 T315I cells were treated with ABL TKIs and/or alisertib for 24 h. Total cell lysates were evaluated by immunoblotting. ^*^P < 0.05. Results represent the mean of three independent experiments.

ABL TKIs are a standard treatment for Ph+ leukemia patients. To investigate the efficacy of ABL TKIs and Aurora kinase inhibitor, Ph+ cells were treated with the ABL TKIs imatinib, nilotinib or ponatinib alone or in combination with alisertib. Co-treatment with imatinib, nilotinib, or ponatinib with alisertib had a synergistic effect that was more potent than the treatment with a single drug (Supplementary Figure 2A–2D). Cytotoxicity and caspase 3/7 activity were also increased by ABL TKI and alisertib treatment (Figure 2A, 2B). Immunoblot analysis revealed that imatinib or ponatinib and alisertib treatment increased caspase 3 and PARP activity and reduced Crk-L phosphorylation in K562 and Ba/F3 T315I cells (Figure 2C). These results indicate that the combination of ABL TKIs and Aurora kinase inhibitor is effective against Ph+ leukemia cells, including those with the T315I mutation.

### Alisertib induces cellular senescence in Ph+ cells

**Figure 3:**
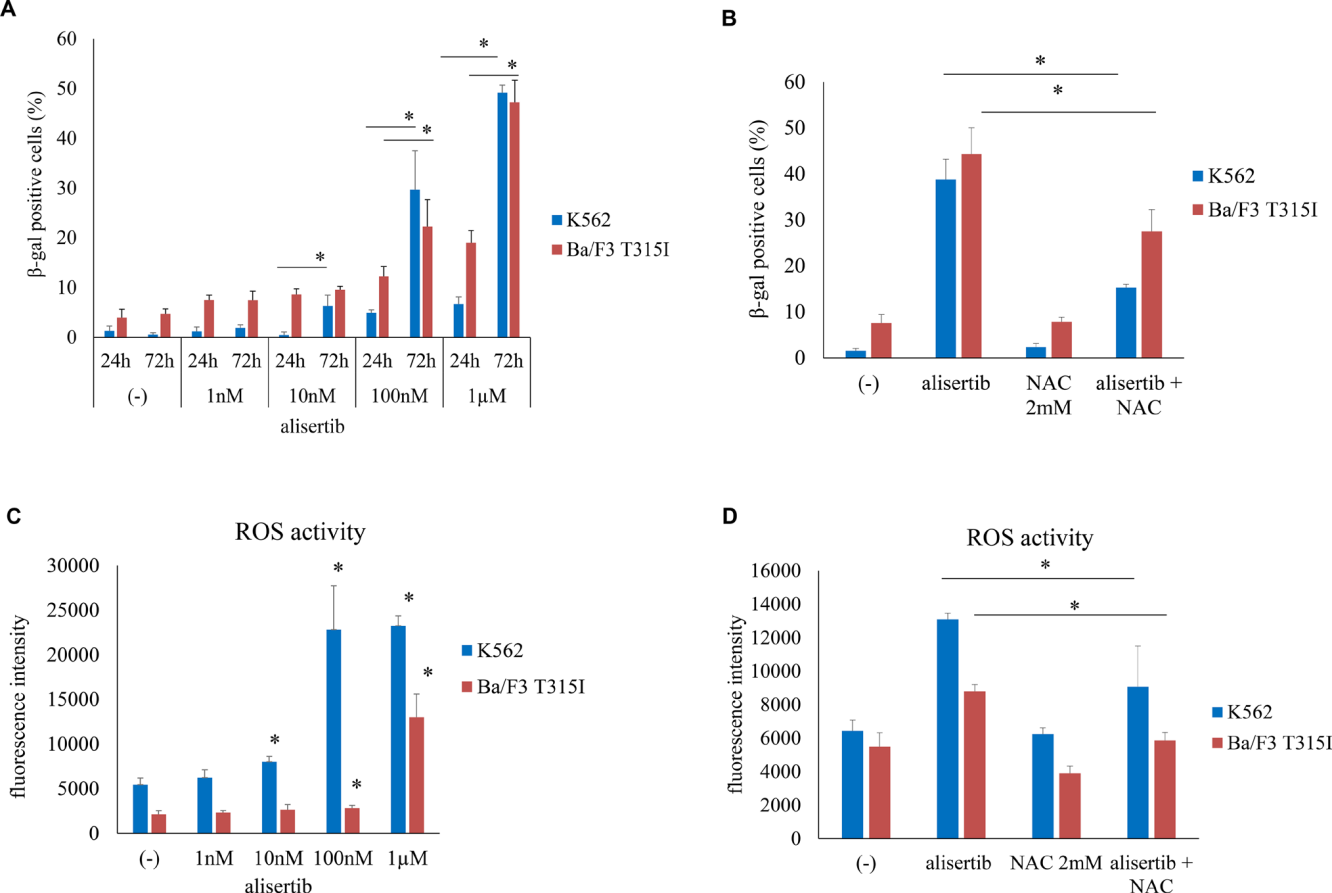
Alisertib induces senescence in Ph+ cells (**A**) K562 or Ba/F3 T315I cells were treated with alisertib for 24 or 72 h; senescence was evaluated by SA-β-gal staining. (**B**) K562 or Ba/F3 T315I cells were treated with alisertib and/or NAC for 72 h; the number of β-gal-positive cells was quantified. (**C**, **D**) K562 or Ba/F3 T315I cells were treated with alisertib and/or NAC for 72 h and intracellular ROS levels were analyzed. ^*^P < 0.05. Results represent the mean of three independent experiments.

Senescence is a terminal cellular outcome that has a cytostatic effect [15]. To determine whether alisertib induces cellular senescence, we examined SA-β-gal activity in K562 and Ba/F3 T315I cells. SA-β-gal staining was increased by alisertib starting on day 1; after 72 h of treatment, the number of SA-β-gal-positive cells was increased in a dose-dependent manner (Figure 3A, Supplementary Figure 3A), an effect that was attenuated in the presence of N-acetyl-l-cysteine (NAC) (Figure 3B, Supplementary Figure 3B), a nonspecific ROS scavenger [16]. ROS can cause premature senescence and induce apoptosis; we found that intracellular ROS levels in Ph+ cells were dose-dependently increased by alisertib treatment (Figure 3C). NAC and synthetic antioxidants abrogated this effect (Figure 3D).

### Aurora A silencing increases ABL TKI activity against Ph+ cells

**Figure 4:**
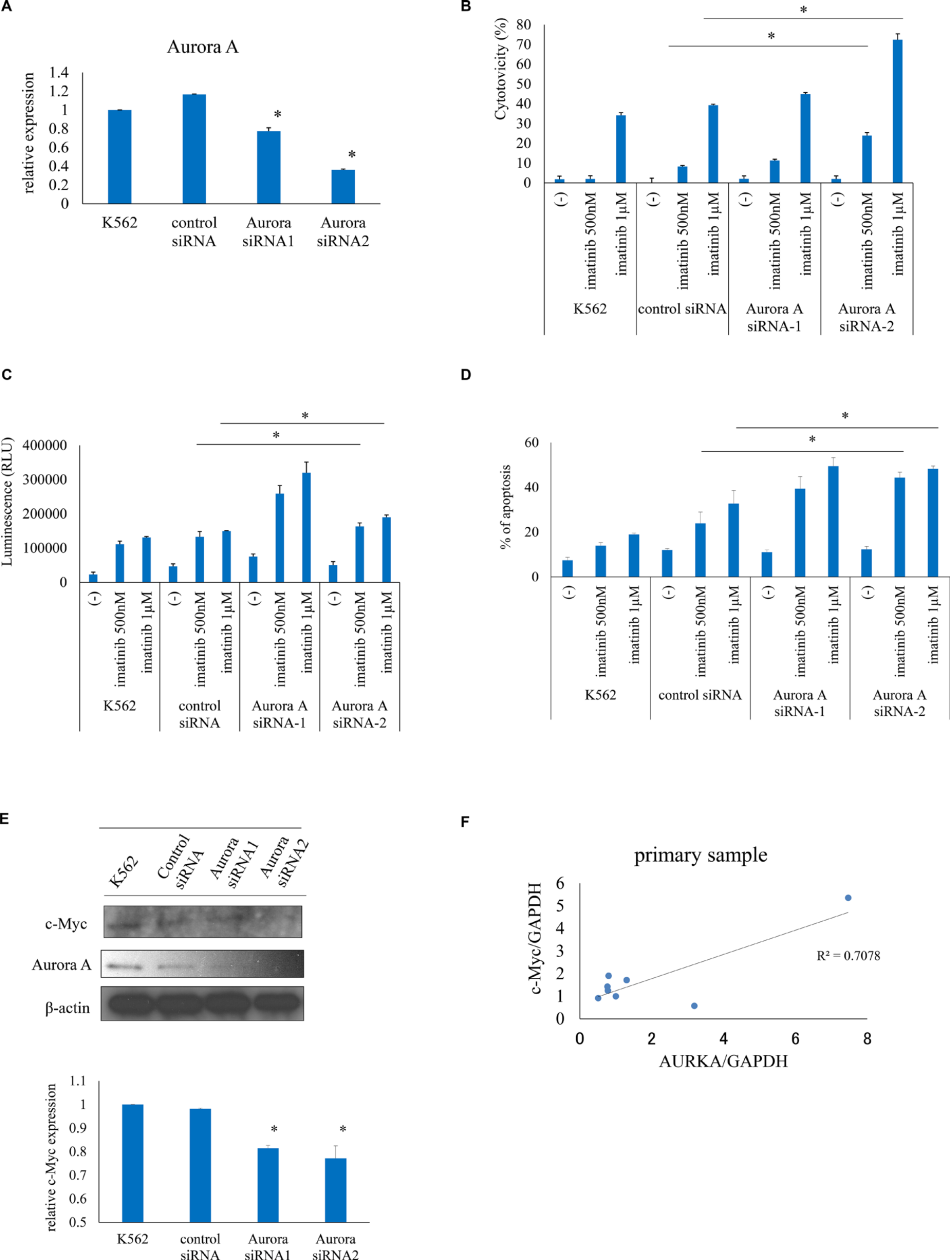
Aurora A knockdown increases ABL TKI activity against Ph+ cells (**A**) Aurora A expression was evaluated by real-time PCR. (**B**–**D**) Effects of Aurora A silencing on cytotoxicity (B), caspase activity (C), and apoptosis (D) induced by imatinib were examined. (**E**) c-Myc expression was analyzed using immunoblotting and quantified. (**F**) Correlation between c-Myc and Aurora A levels in clinical CML samples, as determined by reverse transcription PCR. ^*^P < 0.05. Results represent the mean of two independent experiments.

To evaluate the effect of inhibiting of Aurora A kinase on the leukemia cell response to ABL TKIs, we used a siRNA to knock down Aurora A expression (Figure 4A). Aurora A knockdown enhanced imatinib-induced cell death relative to control siRNA-transfected cells, as evidenced by the increase in cytotoxicity, caspase 3/7 activity, and apoptosis (Figure 4B–4D). Aurora kinase A forms a protein complex with c-Myc in liver cancer [17]. Immunoblot analysis revealed that c-Myc expression was reduced upon Aurora A siRNA transfection (Figure 4E). c-Myc and Aurora A expression were found to be correlated in clinical CML samples, as determined by quantitative reverse transcription PCR (Figure 4F). These results indicate that downregulation of Aurora A is associated with leukemia cell death.

### Efficacy of ABL TKI combined with alisertib in a mouse model

**Figure 5:**
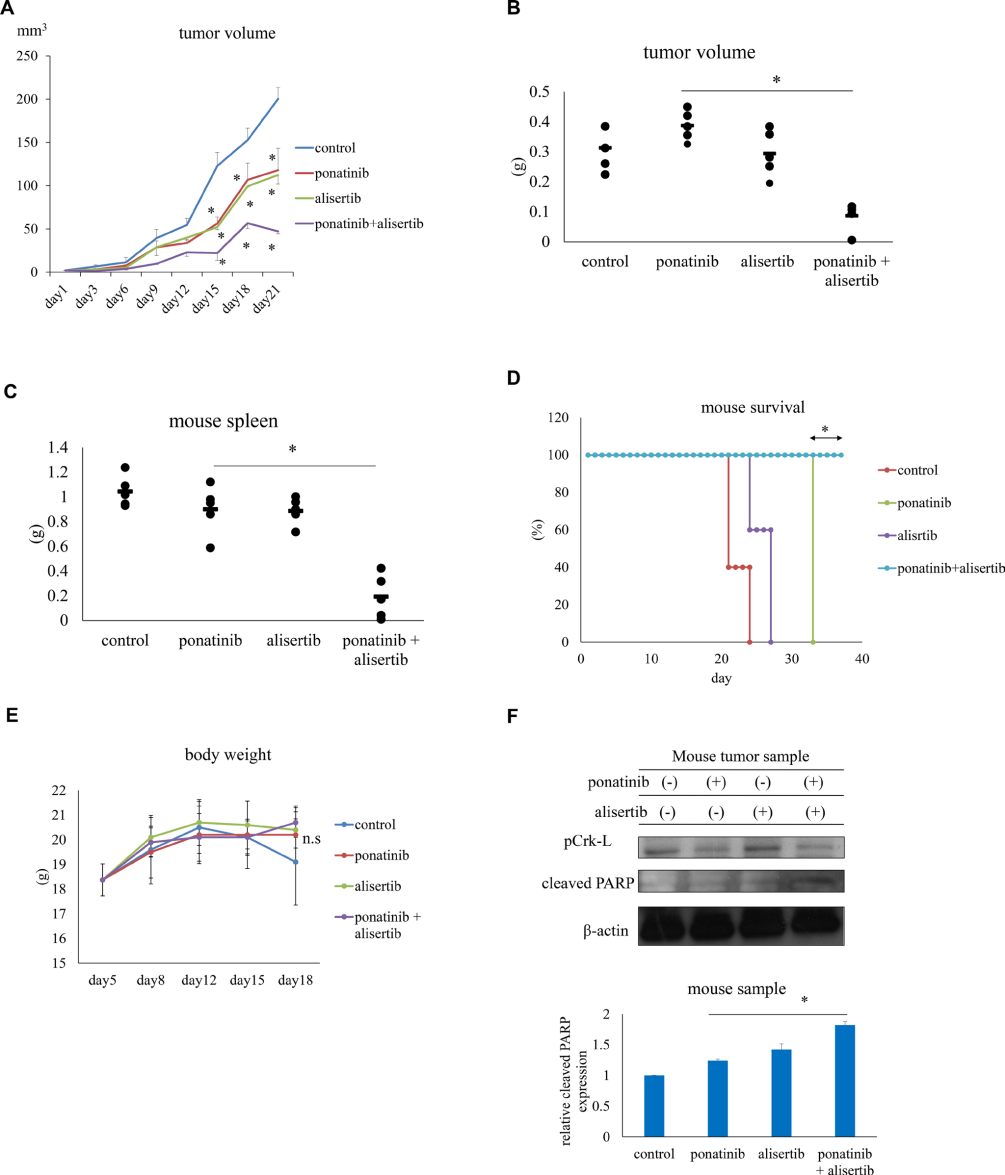
Effects of ponatinib and alisertib on Ba/F3 T315I cell proliferation in a mouse model (**A**) Tumor volumes in mice treated with ponatinib and alisertib (*n* = 5 mice/group). (**B**, **C**) Tumor (B) and spleen (C) volumes in mice with or without ponatinib and alisertib treatment. (**D**) Kaplan–Meier survival curves for ponatinib-and/or alisertib-treated mice subcutaneously injected with Ba/F3 T315I mutant cells. (**E**) Body weight in mice with or without ponatinib and alisertib treatment. (**F**) Immunoblot analysis of p-Crk-L and cleaved-PARP levels in mouse tumor tissue lysates; β-actin served as a loading control. ^*^P < 0.05. Results represent the mean of two independent experiments. n.s., not significant.

To evaluate the efficacy of alisertib *in vivo*, nude mice or NOD-SCID mice were subcutaneously or intraperitoneally inoculated with Ba/F3 T315I cells and the average tumor volume was evaluated every 3 days. We also examined patient-derived xenograft (PDX) models as they are functional to study cancer biology and for drug screening [18]. Ponatinib (20 mg/kg, 5 days/week) or alisertib (30 mg/kg, 5 days/week) administered orally inhibited the growth of Ba/F3 T315I cell tumors *in vivo* to a greater extent than the vehicle control (PBS) (*P* < 0.05) (Figure 5A), whereas co-treatment with alisertib and ponatinib decreased tumor volume (Figure 5B, Supplementary Figure 4A) and spleen size (Figure 5C, Supplementary Figure 4B) to a greater extent than either drug alone. The Kaplan–Meier analysis revealed that co-treatment with ponatinib and alisertib improved survival (Figure 5D). All doses were well tolerated and body weight loss was not observed in the treatment groups (Figure 5E). We also found that Crk-L phosphorylation was reduced and PARP activity was increased after ponatinib and alisertib treatment in mouse tumor samples (Figure 5F). The readout of the flow cytometric analysis of human CD45 positive cells was decreased in the spleen and in the peripheral blood samples by ponatinib and alisertib combination treatment (Supplementary Figure 4C). These results indicate that alisertib increases the efficacy of ABL TKIs and does not have noticeable side effects.

## DISCUSSION

Alisertib is an oral and selective Aurora kinase A inhibitor [19] that functions as an oncoprotein. We showed here that Aurora A is expressed in Ph+ leukemia cells and that inhibiting Aurora A rendered the cells more sensitive to the cytotoxic and pro-apoptotic effects of ABL TKIs. The positive correlation between Aurora A and c-Myc expression in primary CML samples suggests that blocking Aurora kinase activity in combination with ABL TKI treatment can decrease the proliferation and survival of Ph+ cells as a result of c-Myc downregulation.

Cellular senescence is considered a tumor-suppressing mechanism. Our results demonstrated that induction of senescence blocked Ph+ cell growth and increased ROS levels. Alisertib exposure has been linked to an increase in ROS-mediated apoptosis signaling. Thus, ROS play an essential role in alisertib-induced apoptosis in Ph+ cells.

Interestingly, in the mouse xenograft tumor model, co-treatment with ABL TKI, ponatinib, and alisertib prolonged survival and reduced spleen size. Moreover, a combination of 20 mg/kg/day ponatinib and 30 mg/kg/day alisertib suppressed tumor growth as compared to administration of each drug alone. The treatments were well tolerated, suggesting that alisertib combination strategies are clinically feasible. In fact, alisertib and other Aurora kinase inhibitors are now in clinical trial for the treatment of various types of tumors [19–22]. The most common side effects in phase 1 studies were thrombocytopenia, neutropenia, and gastrointestinal toxicity [19]. However, in animal models, alisertib was shown to decrease tumor burden and increase overall survival without toxicity [23]. In the mouse study, ponatinib was more active in the T315I group. The pharmacokinetics of ponatinib, by a phase 1 Japanese study, shows that C_max_ is 86.4 ng/ml in patients treated with 45 mg. These results indicated that ponatinib was more effective in the mouse study possibly because the serum concentration reached a higher value than the *in vitro* treatment dose [24].

In this study, we investigated the efficacy of combination therapy with an ABL TKI and alisertib against Ph+ cells with T315I mutation and ABL TKI resistant cells. Although the underlying mechanisms have yet to be fully defined, our data indicate that Aurora A is an important regulator of tumorigenesis in Ph+ cells. Alisertib induced senescence in Ph+ cells by stimulating ROS production. Thus, Aurora A inhibition is an effective treatment strategy for Ph+ leukemia patients. Moreover, combination therapy with ABL TKI and alisertib exerts a synergistic effect; this combination may improve the clinical outcome of patients with acquired imatinib resistance.

## MATERIALS AND METHODS

### Ethics statement

The present investigation has been conducted in accordance with the ethical standards, according to the Declaration of Helsinki and to national and international guidelines and has been approved by the Tokyo Medical University’s review board.

### Chemicals

Alisertib (MLN8237), Aurora A Inhibitor Ⅰ and ponatinib were purchased from Selleck Chemicals (Houston, TX, USA) and MedKoo Biosciences (Chapel Hill, NC, USA); imatinib was provided by Novartis Pharma AG (Basel, Switzerland). Stock solutions of alisertib and ponatinib were prepared in dimethyl sulfoxide, and imatinib was dissolved in distilled water, aliquoted, and stored at −20° C. Other reagents were obtained from Sigma-Aldrich (St. Louis, MO, USA).

### Cell culture

The K562 Ph+ leukemia cell line was obtained from the American Type Culture Collection (Manassas, VA, USA). T315I-mutant Ba/F3 BCR-ABL cells and ABL TKI resistant K562 cells (K562 IR, K562 NR) were previously established [25, 26]. All cell lines were cultured in Roswell Park Memorial Institute 1640 medium containing 10% fetal bovine serum with or without 500 nM imatinib or nilotinib at 37° C in a humidified atmosphere with 5% CO_2_. Fresh peripheral CML blood samples were collected from patients, and mononuclear cells were isolated using LymphoSepare (Immuno-Biological Laboratories, Okayama, Japan) and either used immediately or cryopreserved in liquid nitrogen until use. The study protocol was approved by the Institutional Review Board of Tokyo Medical University (No. 1974), and written informed consent was obtained from all patients in accordance with the Declaration of Helsinki.

### Cell viability assays

Ph+ leukemia cells were treated with alisertib alone or in combination with imatinib or ponatinib and viability was evaluated by Trypan Blue exclusion or with Cell Counting Kit (Dojindo Laboratories, Kumamoto, Japan) followed by measurement of absorbance at 450 nm. The experiments were performed with triplicate samples.

### Cytotoxicity assay

Cells were treated with various concentration of alisertib and/or imatinib or ponatinib. Cytotoxicity was evaluated based on lactose dehydrogenase (LDH) release using the Cytotoxicity LDH Assay kit with water-soluble tetrazolium [WST] salt (Dojindo Laboratories) according to the manufacturer’s protocol. The amount of LDH released form dead cells was measured using an EnSpire Multimode Plate Reader (PerkinElmer, Waltham, MA, USA).

### Caspase activity

Caspase activity in leukemia cells was examined using the Caspase Glo 3/7 assay kit (Promega, Madison, WI, USA) according to the manufacturer’s protocol. Absorbance was measured on a microplate reader.

### Apoptosis assay

Apoptosis was assessed with the Annexin V detection kit (BD Biosciences, Franklin Lakes, NJ, USA) according to the manufacturer’s protocol. Cells were analyzed by flow cytometry on a FACS Verse instrument (BD Biosciences).

### Detection of reactive oxygen species (ROS)

Cells were treated with indicated concentration of alisertib and/or ABL TKIs for 72 h. Intracellular ROS levels were analyzed with the 2’,7’-dichlorofluorescein diacetate (DCFDA)/H2DCFDA Cellular Reactive Oxygen Species Detection Assay kit (Abcam, Cambridge, UK) according to the manufacturer’s protocol. Fluorescence was measured on a microplate reader.

### β-Galactosidase (β-gal) staining

Senescence-associated (SA)-β-gal staining was used to evaluate senescence in Ph+ cells. SA-β-gal activity was qualitatively assessed with a kit (Cell Signaling Technology, Danvers, MA, USA) according to the manufacturer’s instructions along with examination on an inverted microscope (Olympus, Tokyo, Japan). The percentage of senescent cells was counted as the number of SA-β-gal-positive cells per total number of cells.

### Cell cycle analysis

Cell cycle status was analyzed with the BD Cycletest Plus DNA Reagent kit (BD Biosciences) according to the manufacturer’s protocol. After 48 h of treatment with alisertib, K562 and Ba/F3 T315I cell cycle distribution was analyzed by flow cytometry.

### Immunoblot analysis

Immunoblotting was performed according to a previously described method [27, 28]. Briefly, after incubation in indicated concentrations of inhibitor, cells were washed with ice-cold phosphate-buffered saline (PBS) and lysed in radioimmunoprecipitation lysis buffer. The protein content of lysates was determined with a kit (Bio-Rad, Hercules, CA, USA) and proteins were resolved by polyacrylamide gel electrophoresis and transferred to a polyvinylidene difluoride membrane (Millipore, Billerica, MA, USA) that was incubated with primary antibodies at the appropriate dilution for 1 h. The blots were then probed with the secondary antibodies and developed with an enhanced chemiluminescence system (Amersham Pharmacia Biotech, Little Chalfont, UK). Antibodies against the following proteins were used: phosphorylated (p-)ABL, p-Crk-L, cleaved caspase 3, cleaved poly (ADP-ribose) polymerase (PARP), and p-Aurora, p-Aurora A (Thr288)/Aurora B (Thr232)/Aurora C (Thr198) (all from Cell Signaling Technology); Crk-L (Millipore); Aurora A (GeneTex, Irvine, CA, USA); and ABL, c-Myc, and β-actin (Santa Cruz Biotechnology, Santa Cruz, CA, USA). Three independent experiments were performed. Protein band intensity was evaluated using ImageJ software (National Institutes of Health, Bethesda, MD, USA).

### Small interfering (si)RNA transfection

siRNA targeting Aurora A was purchased from Sigma-Aldrich. K562 cells were transfected with Aurora A or control siRNA by electroporation as previously described [29].

### Real-time PCR

Total RNA was extracted from cultured cells using the RNeasy Mini kit (Qiagen, Venlo, Netherlands) and subjected to DNase digestion with the RNase-Free DNase Set (Qiagen). Reverse transcription was performed using the First Strand cDNA Synthesis kit (OriGene Technologies, Rockville, MD, USA). Real-time reverse transcription PCR was performed using FastStart Essential DNA Green Master Mix according to the recommended protocol on a LightCycler 96 system (Roche, Mannheim, Germany). Primers were purchased from Takara Bio, Otsu, Japan), and β-actin or glyceraldehyde 3-phosphate dehydrogenase served as an internal control.

### *In vivo* assay

Mice were maintained under specific pathogen-free conditions. Experiments were performed with the approval of the Institutional Animal Care and Use Committee of Tokyo Medical University. Female mice BALB/c-nu/nu mice or NOD-SCID mice (6 weeks old) were subcutaneously or intraperitoneally injected with 1 × 10^7^ Ba/F3 T315I cells or T315I positive human samples, and then orally administered 20 mg/kg ponatinib, 30 mg/kg alisertib, or both for 5 days/week. Control mice were administered PBS by the same route. At predetermined time points, tumor size and mouse survival were recorded. The average tumor weight and spleen size per mouse were calculated and used to determine mean tumor volume or weight ± SEM (*n* = 5) for each group. Mouse spleen, tumor cells, and peripheral blood samples were collected at indicated time points, and the samples were also analyzed by flow cytometry.

### Statistical analysis

A Student’s *t*-test and two-way analysis of variance were used to compare the means of drug treatment and control groups. *P* < 0.05 was considered statistically significant.

## SUPPLEMENTARY MATERIALS FIGURES



## Abbreviations

ABL: Abelson murine leukemia viral oncogene homolog
TKIs: tyrosine kinase inhibitors
CML: chronic myeloid leukemia
Ph+: Philadelphia chromosome-positive
BCR-ABL1: breakpoint cluster region-Abelson murine leukemia viral oncogene homolog 1
NAC: N-acetyl-l-cysteine
ROS: reactive oxygen species
DCFDA: 2’,7’-dichlorofluorescein diacetate
β-gal: β-galactosidase
SA: senescence-associated
PBS: phosphate-buffered saline
PARP: poly (ADP-ribose) polymerase
siRNA: small interfering RNA
