# Supplementary material for: Therapeutic targeting of Aurora A kinase in Philadelphia chromosome-positive ABL tyrosine kinase inhibitor-resistant cells
Source: Oncotarget. 2018 Aug 21;9(65):32496–506. doi: 10.18632/oncotarget.25985 (PMC6126699; doi:10.18632/oncotarget.25985)
Supplement: Supplementary file 1 [file oncotarget-09-32496-s001.pdf]

Therapeutic targeting of Aurora A kinase in Philadelphia chromosome-positive ABL tyrosine kinase inhibitor-resistant cells

SUPPLEMENTARY MATERIALS

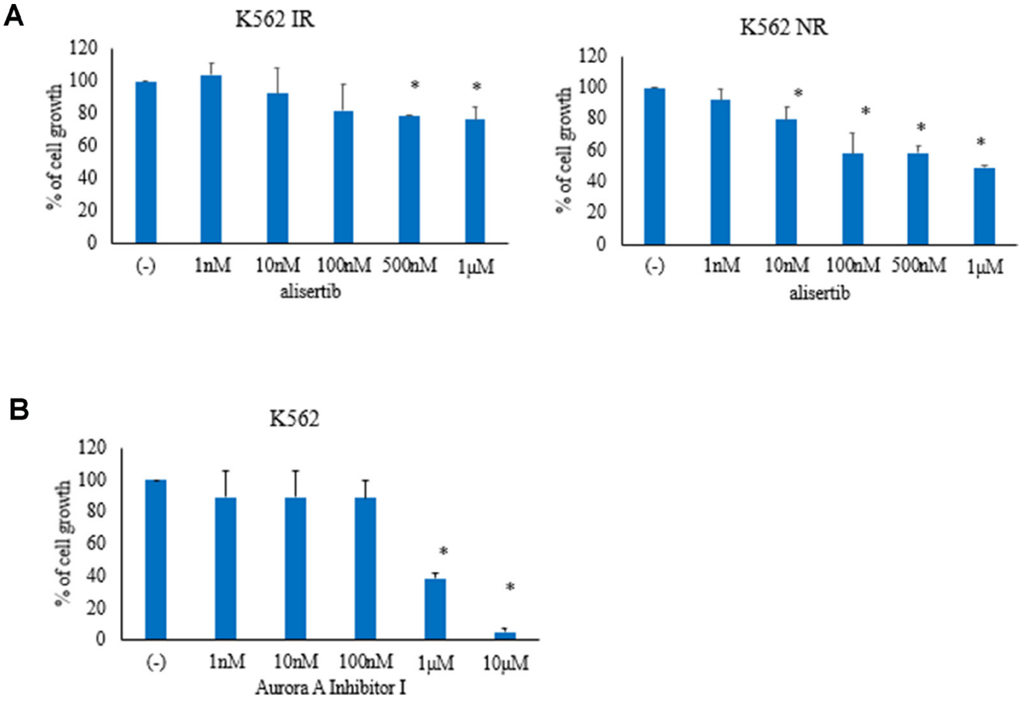

Supplementary Figure 1: Efficacy of alisertib and Aurora A inhibitor against Ph+ cells. (**A**) ABL TKI resistant K562 cells (K562 IR, NR) were treated with indicated concentrations of alisertib for 72 h and cell growth was evaluated. (**B**) K562 cells were treated with indicated concentrations of Aurora A Inhibitor Ⅰ for 72 h and cell growth was evaluated. Data are representative of three independent sets of experiments. $^{*}P < 0.05$.

A

K562		imatinib							
		(-)	2.4nM	9.7nM	39nM	156nM	625nM	2.5µM	10µM
alisertib	(-)	0	24	19	20	68	96	96	100
	2.4nM	7	6	13	19	67	96	97	100
	9.7nM	23	23	24	35	66	96	97	99
	39nM	77	75	80	85	95	98	99	100
	156nM	83	80	82	91	97	99	99	100
	625nM	87	87	88	93	96	99	99	100
	2.5µM	93	92	92	94	97	99	99	100
	10µM	93	93	94	94	96	98	98	100

B

Ba/F3 T315I		ponatinib							
		(-)	0.24nM	0.97nM	3.9nM	15.6nM	62.5nM	250nM	1µM
alisertib	(-)	0	0	0	35	93	100	99	99
	2.4nM	26	17	20	49	93	99	99	100
	9.7nM	2	21	13	57	95	99	99	100
	39nM	18	14	29	57	95	99	99	100
	156nM	62	61	63	79	99	99	100	99
	625nM	90	90	90	96	99	100	100	100
	2.5µM	91	93	92	95	100	100	100	100
	10µM	95	96	96	99	100	100	100	100

C

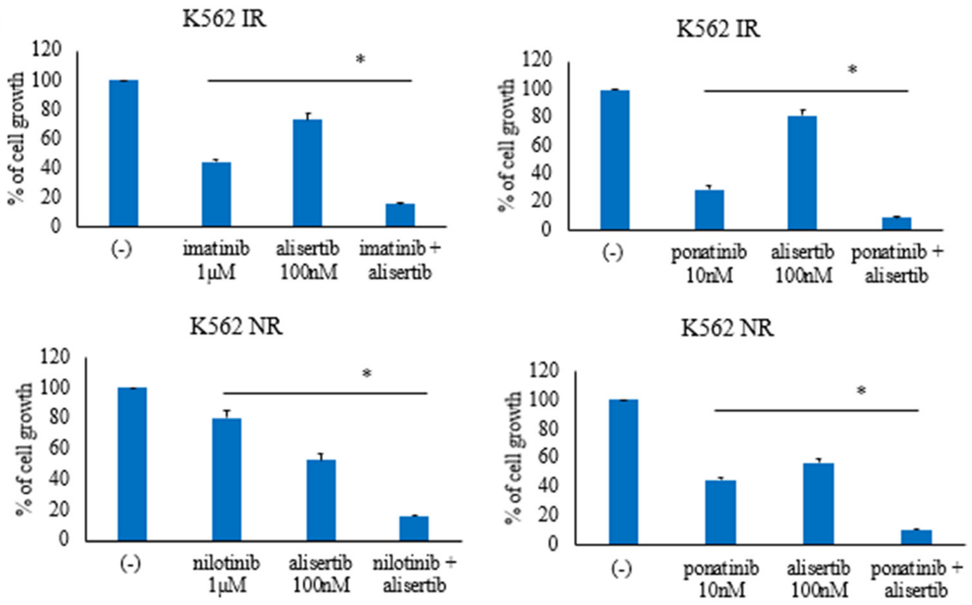

D

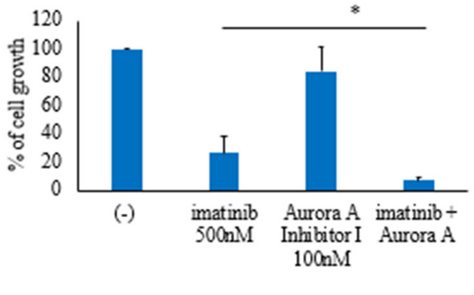

Supplementary Figure 2: Efficacy of ABL TKIs and alisertib against Ph+ cells. (**A–C**) K562, Ba/F3 T315I, K562 IR, K562 NR cells were treated with indicated concentrations of imatinib, nilotinib, ponatinib, and/or alisertib for 72 h. Cell growth relative to dimethyl sulfoxide (DMSO)-treated cells was evaluated. Data are representative of three independent sets of experiments. (**D**) K562 cells were treated with indicated concentrations of imatinib and/or Aurora A Inhibitor Ⅰ for 72 h and cell growth was evaluated. $^{*}P < 0.05$.

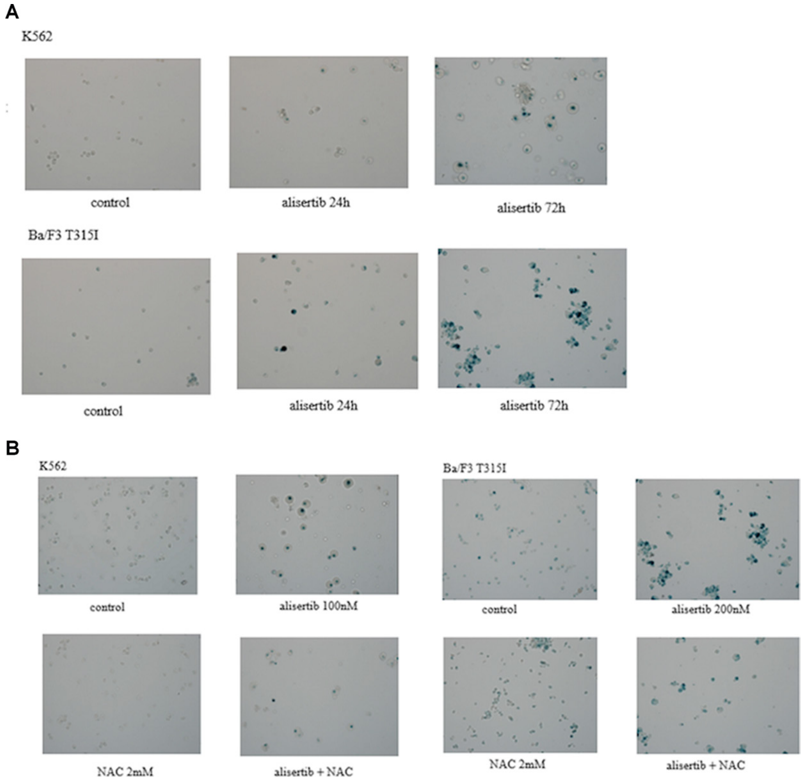

Supplementary Figure 3: Alisertib induces senescence in Ph+ cells. (**A**) K562 or Ba/F3 T315I cells were treated with alisertib for 24 or 72 h; senescence was evaluated. Magnification: 100×. (**B**) K562 or Ba/F3 T315I cells were treated with alisertib and/or NAC for 72 h; senescence was evaluated. Magnification: 100×.

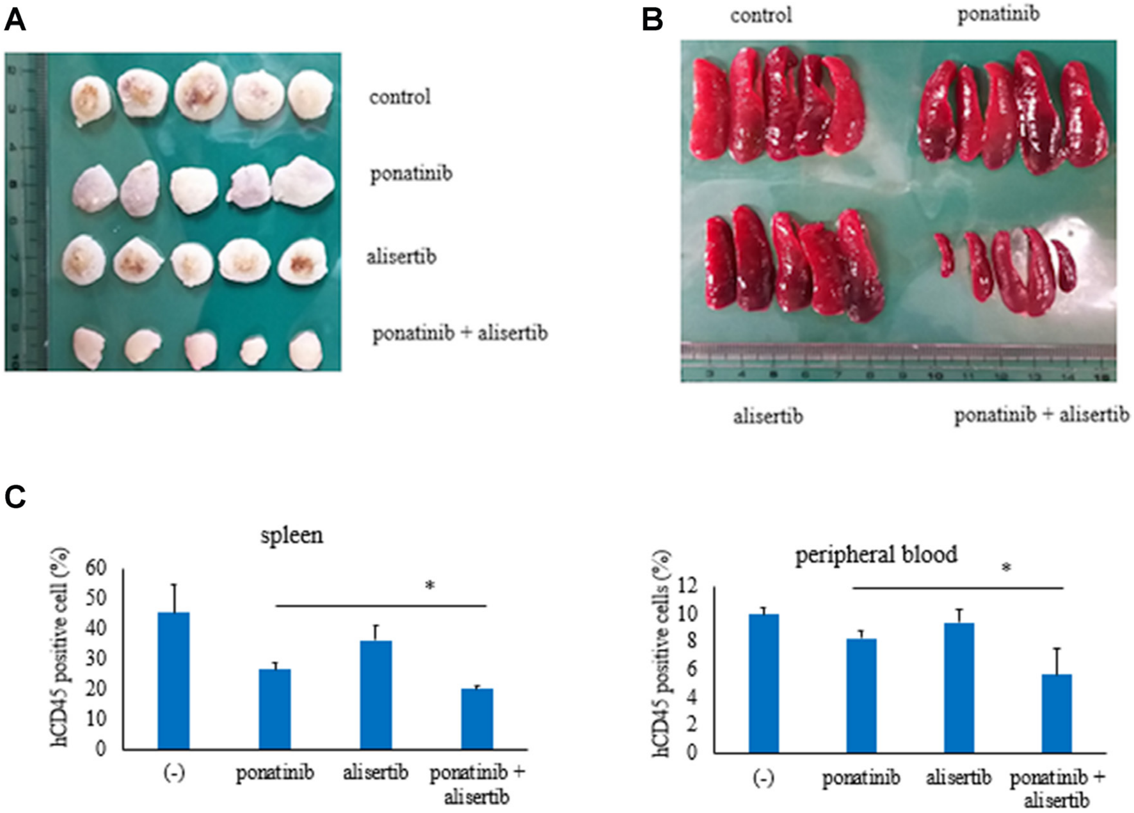

Supplementary Figure 4: Effects of ponatinib and alisertib on Ba/F3 T315I cell proliferation in a mouse model. (**A**, **B**) Tumor (A) and spleen (B) volumes in mice with or without ponatinib and alisertib treatment. (**C**) Mouse spleen, and peripheral blood samples were collected, and human CD 45 cells were analyzed by flow cytometry. $^{*}P < 0.05$.
